# Non-invasive Assessment of Neurovascular Coupling After Aneurysmal Subarachnoid Hemorrhage: A Prospective Observational Trial Using Retinal Vessel Analysis

**DOI:** 10.3389/fneur.2021.690183

**Published:** 2021-06-14

**Authors:** Walid Albanna, Catharina Conzen, Miriam Weiss, Katharina Seyfried, Konstantin Kotliar, Tobias Philip Schmidt, David Kuerten, Jürgen Hescheler, Anne Bruecken, Arno Schmidt-Trucksäss, Felix Neumaier, Martin Wiesmann, Hans Clusmann, Gerrit Alexander Schubert

**Affiliations:** ^1^Department of Neurosurgery, RWTH Aachen University, Aachen, Germany; ^2^Institute for Neurophysiology, University of Cologne, Cologne, Germany; ^3^Department of Medical Engineering and Technomathematics, FH Aachen University of Applied Sciences, Aachen, Germany; ^4^Department of Ophthalmology, RWTH Aachen University, Aachen, Germany; ^5^Department of Intensive Care and Intermediate Care, RWTH Aachen University, Aachen, Germany; ^6^Department of Exercise and Health Sciences, University of Basel, Basel, Switzerland; ^7^Department of Diagnostic and Interventional Neuroradiology, RWTH Aachen University, Aachen, Germany

**Keywords:** aneurysmal subarachnoid hemorrhage, cerebral infarction, delayed cerebral ischemia, microvascular function, neurovascular coupling, retinal vessel analysis

## Abstract

**Objective:** Delayed cerebral ischemia (DCI) is a common complication after aneurysmal subarachnoid hemorrhage (aSAH) and can lead to infarction and poor clinical outcome. The underlying mechanisms are still incompletely understood, but animal models indicate that vasoactive metabolites and inflammatory cytokines produced within the subarachnoid space may progressively impair and partially invert neurovascular coupling (NVC) in the brain. Because cerebral and retinal microvasculature are governed by comparable regulatory mechanisms and may be connected by perivascular pathways, retinal vascular changes are increasingly recognized as a potential surrogate for altered NVC in the brain. Here, we used non-invasive retinal vessel analysis (RVA) to assess microvascular function in aSAH patients at different times after the ictus.

**Methods:** Static and dynamic RVA were performed using a Retinal Vessel Analyzer (IMEDOS Systems GmbH, Jena) in 70 aSAH patients during the early (d_0−4_), critical (d_5−15_), late (d_16−23_) phase, and at follow-up (f/u > 6 weeks) after the ictus. For comparison, an age-matched cohort of 42 healthy subjects was also included in the study. Vessel diameters were quantified in terms of the central retinal arterial and venous equivalent (CRAE, CRVE) and the retinal arterio-venous-ratio (AVR). Vessel responses to flicker light excitation (FLE) were quantified by recording the maximum arterial and venous dilation (MAD, MVD), the time to 30% and 100% of maximum dilation (tMAD_30_, tMVD_30_; tMAD, tMVD, resp.), and the arterial and venous area under the curve (AUC_art_, AUC_ven_) during the FLE. For subgroup analyses, patients were stratified according to the development of DCI and clinical outcomes after 12 months.

**Results:** Vessel diameter (CRAE, CRVE) was significantly smaller in aSAH patients and showed little change throughout the whole observation period (*p* < 0.0001 vs. control for all time periods examined). In addition, aSAH patients exhibited impaired arterial but not venous responses to FLE, as reflected in a significantly lower MAD [2.2 (1.0–3.2)% vs. 3.6 (2.6–5.6)% in control subjects, *p* = 0.0016] and AUC_art_ [21.5 (9.4–35.8)%^*^s vs. 51.4 (32.5–69.7)%^*^s in control subjects, *p* = 0.0001] on d_0−4_. However, gradual recovery was observed during the first 3 weeks, with close to normal levels at follow-up, when MAD and AUC_art_ amounted to 3.0 [2.0–5.0]% (*p* = 0.141 vs. control, *p* = 0.0321 vs. d_5−15_) and 44.5 [23.2–61.1]%^*^s (*p* = 0.138 *vs*. control, *p* < 0.01 vs. d_0−4_ & d_5−15_). Finally, patients with clinical deterioration (DCI) showed opposite changes in the kinetics of arterial responses during early and late phase, as reflected in a significantly lower tMAD_30_ on d_0−4_ [4.0 (3.0–6.8) s vs. 7.0 (5.0–8.0) s in patients without DCI, *p* = 0.022) and a significantly higher tMAD on d_16−23_ (24.0 (21.0–29.3) s *vs*. 18.0 (14.0–21.0) s in patients without DCI, *p* = 0.017].

**Conclusion:** Our findings confirm and extend previous observations that aSAH results in sustained impairments of NVC in the retina. DCI may be associated with characteristic changes in the kinetics of retinal arterial responses. However, further studies will be required to determine their clinical implications and to assess if they can be used to identify patients at risk of developing DCI.

**Trial Registration:** ClinicalTrials.gov Identifier: NCT04094155.

## Introduction

Aneurysmal subarachnoid hemorrhage (aSAH) due to sudden rupture of a cerebral aneurysm is responsible for only 5–7% of all stroke events (1) but is associated with a fatality rate of almost 50%, accounting for up to 20% of all cerebrovascular-related deaths (2). Owing to a complex pathophysiology that involves early and delayed forms of brain injury, a significant number of patients surviving aSAH remain disabled with or without compromise detected on detailed neuropsychological testing. Only 10% of patients recover completely. The most common complication responsible for long-term impairments is delayed cerebral ischemia (DCI), which is typically observed during the first two or at most 3 weeks after aSAH onset and is associated with the appearance of a new focal neurologic deficit or a persistent decline in the patient's Glasgow Coma Scale score (3). Although traditionally attributed to vasoconstriction of large caliber proximal vessels (i.e., angiographic vasospasm) secondary to degradation of subarachnoid blood and intrathecal immune activation, more recent findings also point to a critical role of microvascular dysfunction (4–7). In particular, several studies performed in animal models have demonstrated a progressive impairment and partial inversion of cerebral neurovascular coupling (NVC) (8–10), the process responsible for adjusting local cerebral blood flow (CBF) to regionally heterogeneous changes in metabolic demand due to neuronal activation. While there is evidence for similar impairment in human aSAH patients (11–13), clinical assessment of NVC remains a diagnostic challenge that is usually achieved through invasive CBF measurements or highly specialized and expensive functional imaging techniques. An alternative approach to evaluate NVC is to measure microvascular responses in the retina, an embryological part of the central nervous system that can be assessed non-invasively by retinal vessel analysis (RVA). Because cerebral and retinal microvasculature bear a close anatomical correlation and are governed by common regulatory mechanisms, retinal vascular changes are increasingly recognized as potential screening option for altered NVC in the brain (14, 15). For example, a potential role of RVA for predicting cerebrovascular changes has previously been shown in elderly adults (16, 17), after coarctation repair (18) and in patients with diabetes (19) or Alzheimer's disease (20). During aSAH, the sudden rise of intracranial pressure often forces subarachnoid blood into the pre-retinal space (21), which may expose retinal micro-vessels to the same blood degradation products and inflammatory cytokines thought to be responsible for microvascular dysfunction in the brain. In addition, recent discovery of a cerebral and retinal glymphatic system (22) argues for the existence of perivascular pathways through which vasoactive agents formed in the subarachnoid space may also directly reach the retinal microvasculature. We have previously demonstrated the feasibility of RVA in aSAH patients (23) and provided first evidence for retinal vasoconstriction and signs of impaired NVC in these patients compared to healthy control subjects (11). In this prospective study on a larger cohort of aSAH patients, we sought to validate our previous findings and evaluate the relationship between potential changes in NVC as detected by RVA and the occurrence of DCI and long-term clinical outcome.

## Materials and Methods

Patients with aSAH admitted to the Department of Neurosurgery at the RWTH Aachen University Hospital between July 2015 and December 2019 were considered for enrollment into the present study. Patients were selected based on the following inclusion criteria: (1) age ≥ 18 years, (2) aneurysmal origin of SAH as confirmed by computed tomography (CT) angiography and/or digital subtraction angiography (DSA), (3) absence of intraocular comorbidities or contraindications that would have precluded application of mydriatic agents, and (4) absence of Terson syndrome unless it did not interfere with the analysis of selected retinal vessels. Before enrolment, written informed consent according to the Declaration of Helsinki was obtained from all patients or their legal representatives and the study protocol was approved by the local Research Ethics Committee of the RWTH Aachen University (EK 069/15).

A total of 70 patients with aSAH were prospectively recruited, in whom measurements were performed 0–4 days (early phase: d_0−4_), 5–15 days (critical phase: d_5−15_), 16–23 days (late phase: d_16−23_) and more than 6 weeks (follow-up: f/u) after the ictus. If more than one measurement was available for a given patient in a given phase, all measurements in this phase were averaged to calculate a mean valued. For comparison, an age-matched cohort of 42 healthy subjects who were measured by RVA at the Department of Exercise and Health Sciences at the University of Basel was also included in the study.

The clinical severity of aSAH on admission was assessed according to the Hunt and Hess (HH) grading scale and the degree of aneurysmal bleeding was scored according to the modified Fischer (mFS) scale based on CT scans. Patients were treated according to current multidisciplinary consensus guidelines (3, 24, 25). The aneurysm was secured by neurosurgical clipping or endovascular coiling based on an interdisciplinary consensus. Patients received prophylactic nimodipine therapy (180–360 mg per day) unless precluded by hemodynamic instability. All patients were continuously monitored for complications such as delayed cerebral ischemia (DCI), which was considered as a neurological worsening according to the definition by Vergouwen et al. (3) or territorial or watershed hypoperfusion on perfusion CT scans. In analgosedated patients, deterioration in cerebral microdialysis (71 high cut-off catheter, μdialysis, Stockholm, Sweden) or cerebral tissue oxygen saturation (Neurovent PTO, Raumedic AG, Helmbrechts, Germany) were used as surrogates to trigger CT perfusion. First-tier treatment in case of DCI consisted of induced hypertension (>180 mmHg systolic blood pressure) as per institutional protocol. In refractory cases without clinical or neuromonitoring improvement but confirmed misery perfusion on CTA or DSA, second-tier rescue therapy by balloon dilatation and/or continuous intra-arterial infusion of nimodipine was considered based on angiographic findings (26, 27). Clinical outcome after 12 months according to the extended Glasgow Outcome Scale (GOS-E) was assessed by an independent investigator based on a telephone interview, clinical investigation in the outpatient clinic or clinical status compiled from the medical reports. A summary of the demographic and clinical data obtained on admission or during in-hospital treatment is provided in Table 1.

**Table 1:** Demographic and clinical data of control and aSAH patients.

**Patient characteristic**	**Patients, *n* (%)**	**Control, *n* (%)**
All patients	70 (100%)	42 (100%)
**Gender**		
Female	51 (73%)	20 (48%)
Male	19 (27%)	22 (52%)
**Age**		
	51 [48–59] yrs	50 [43–59] yrs
**Aneurysm location**		
Anterior circulation	53 (76%)	
Posterior circulation	16 (23%)	
Both	1 (1%)	
**Hunt and hess (HH) grade**		
Good grade (HH_1−3_)	56 (80%)	
Poor grade (HH_4−5_)	14 (20%)	
**Modified fisher (mFS) scale**		
Thin blood (mFS_0−2_)	34 (49%)	
Thick blood (mFS_3−4_)	36 (51%)	
**Treatment modality**		
Clipping	24 (34%)	
Coiling	44 (63%)	
Both	2 (3%)	
**Delayed cerebral ischemia (DCI)**		
DCI	23 (32%)	
DCI-related infarction	1 (1%)	
**Outcome**		
Favorable (GOS-E_5−8_)	64 (92%)	
Unfavorable (GOS-E_1−4_)	6 (8%)	

### Retinal Vessel Analysis

**Figure 1:**
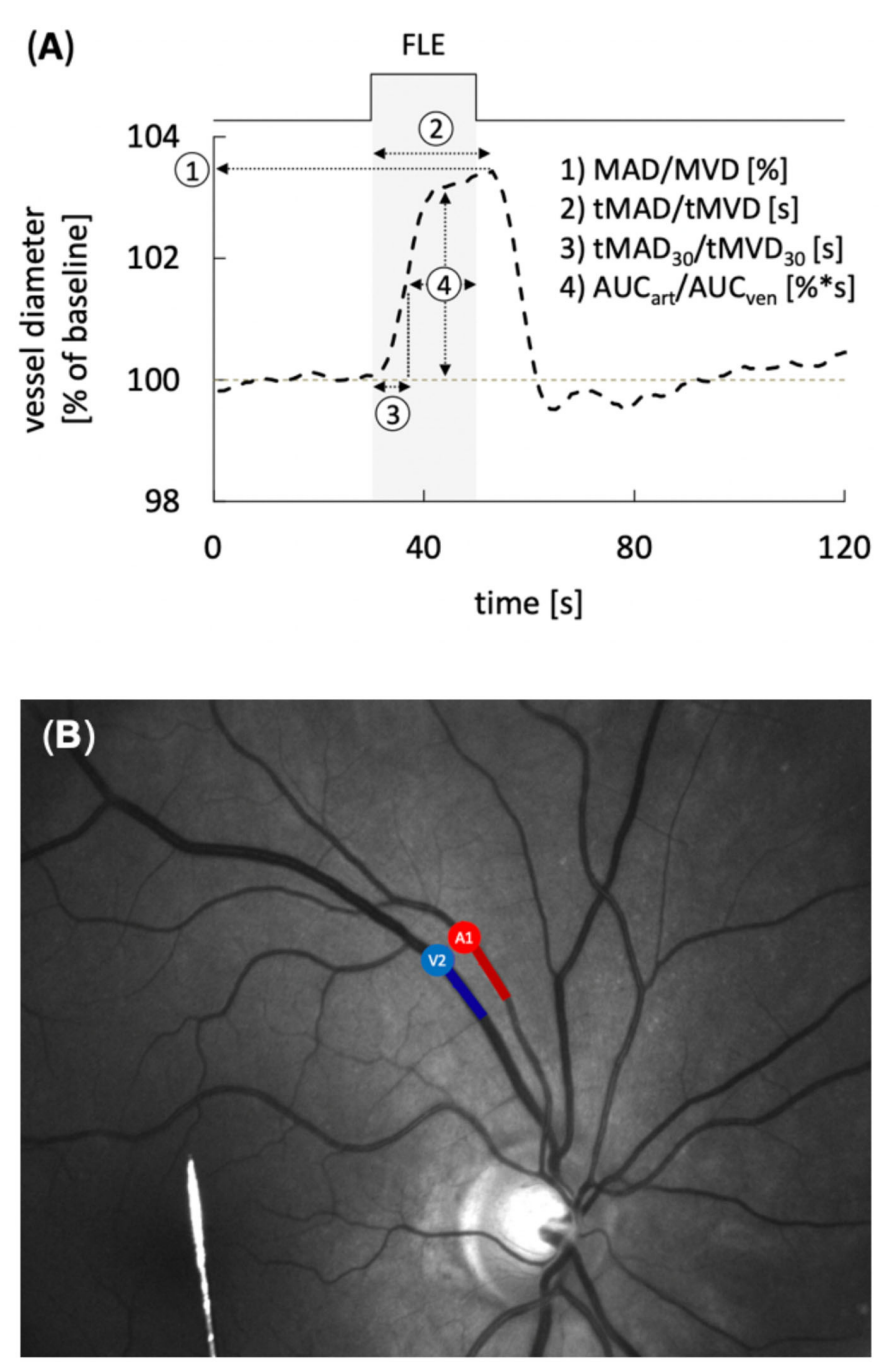
Static and dynamic retinal vessel analysis (RVA). **(A)** Average retinal vessel response to flicker light excitation (FLE) in healthy subjects with the different parameters determined for dynamic RVA. The stimulation period is indicated above and by light gray shading. Parameters used for quantification of the response comprised maximum arterial or venous dilation (MAD/MVD), time to maximum arterial or venous dilation (tMAD/tMVD) relative to flicker initiation, time to 30% of the maximum arterial or venous dilation (tMAD_30_/tMVD_30_) relative to flicker initiation, and arterial or venous area under the curve (AUC_art_/AUC_ven_) during the FLE. **(B)** Example of a monochromatic fundus image used for RVA. Arterial (A) and venous (V) segments are indicated in red and blue, respectively.

Static and dynamic retinal vessel analyses were performed using a maneuverable Retinal Vessel Analyzer (IMEDOS Systems GmbH, Jena, Germany) as described previously (11, 23). Briefly, after unilateral application of a mydriatic agent (Tropicamide, Mydriaticum Stulln UD, Pharma Stulln GmbH, Stulln, Germany), conscious patients were examined in a sitting position while analgo-sedated patients were examined in supine position with the head and torso partially turned. For assessment of NVC, retinal veins and arteries were focused and the vessel diameters recorded continuously in operator-selected regions of interest located equidistant to the papilla. Measurements were performed with a standard 350 s dynamic vessel analysis protocol comprised of 50 s of baseline recording and three cycles of flicker-light excitation (FLE). For extended analysis the recorded vessel diameters were expressed as percentage of the baseline diameter and plotted as a function of time as described in detail elsewhere (20, 28). To improve the signal-to-noise ratio, the individual response curves obtained for each subject during the three successive cycles of FLE were averaged. In healthy subjects, the typical arterial response curves thus obtained feature primary vasodilation upon initiation of FLE, which reaches a maximum after a characteristic latency and is followed, after termination of the stimulus, by a reflectory vasoconstriction (29). The general shape and amplitude of the response curve provide an index for the integrity of NVC, and were quantified by calculating maximum arterial and venous dilatation (MAD, MVD), the arterial and venous area-under-the-curve (AUC_art_, AUC_ven_) and the time to reach 30% (tMAD_30_, tMVD_30_) and 100% (tMAD, tMVD) of MAD and MVD after flicker initiation (Figure 1A). Following the dynamic analysis, monochromatic fundus images for static retinal vessel analysis (Figure 1B) were obtained at a 30° camera angle with the optic nerve centered and processed using a dedicated software tool (VisualIS, IMEDOS Systems GmbH., Jena, Germany). Central retinal arterial and venous equivalents (CRAE and CRVE, respectively), and arterio-venous ratio (AVR) were calculated as described previously (30, 31). All dynamic and static examinations were routinely paralleled by multimodal monitoring of mean arterial blood pressure (MAP), heart rate, blood gases, oxygenation and intracranial pressure (ICP), ensuring physiological conditions at the time of image acquisition. Measurement was aborted in case of hemodynamic or ventilatory instability.

### Statistical Analysis

Normal distribution was tested using the Kolmogorow-Smirnow normality test. Quantitative parameter values are presented as mean ± standard deviation (SD) or as median [1 quartile−3 quartile], and as percentage in case of nominal data. Differences between two groups were analyzed using the two-sided student *t*-test, Mann–Whitney-test or Chi-Square test, respectively. For subgroup analyses with regard to DCI and outcome, all patients were stratified into groups according to the development of DCI (DCI *vs*. no DCI) and clinical outcome after 12 months (unfavorable: GOS-E_1−4_ *vs*. favorable: GOS-E_5−8_), respectively (see Supplementary Tables 1, 2). For analysis of the influence of nimodipine, all measurements (see Supplementary Table 3) or all patients (see Supplementary Table 4) were stratified into groups according to oral nimodipine treatment (measurements in nimodipine-treated patients *vs*. measurements in patients with no nimodipine treatment for at least 24 h). Statistical significance was set at p < 0.05; statistical results with p < 0.1 were accepted as a trend. All analyses were performed using Excel (Microsoft Office Excel 2010, Californian, USA), SPSS v. 21 (IBM Chicago, Illinois, USA), Numbers®, Apple Inc., Cupertino, USA, GraphPad Prism® and GraphPad Software, Inc., La Jolla, USA.

## Results

The demographic data of patients included in this study are summarized in Table 1 In total, the aSAH cohort comprised 70 patients with a median age of 51 [48–59] years, of whom 51 (73%) were female and 19 (27%) were male. Twenty-three (32%) of the patients developed delayed cerebral ischemia (DCI), which progressed to infarction in one patient. The clinical outcome after 12 months was favorable (GOS-E_5−8_) in 64 (92%) and unfavorable (GOS-E_1−4_) in 6 (8%) of the patients. Retinal vessel analysis (RVA) could be performed in 32, 53, 23, and 40 patients during the early (d_0−4_), critical (d_5−15_), late (d_16−23_) phase, and follow-up, respectively. For comparison, a cohort of 42 healthy subjects with a median age of 50 [43–59] years, of whom 20 (48%) were female and 22 (52%) were male, was also included in the study (Table 1).

### Vessel Diameters

**Figure 2:**
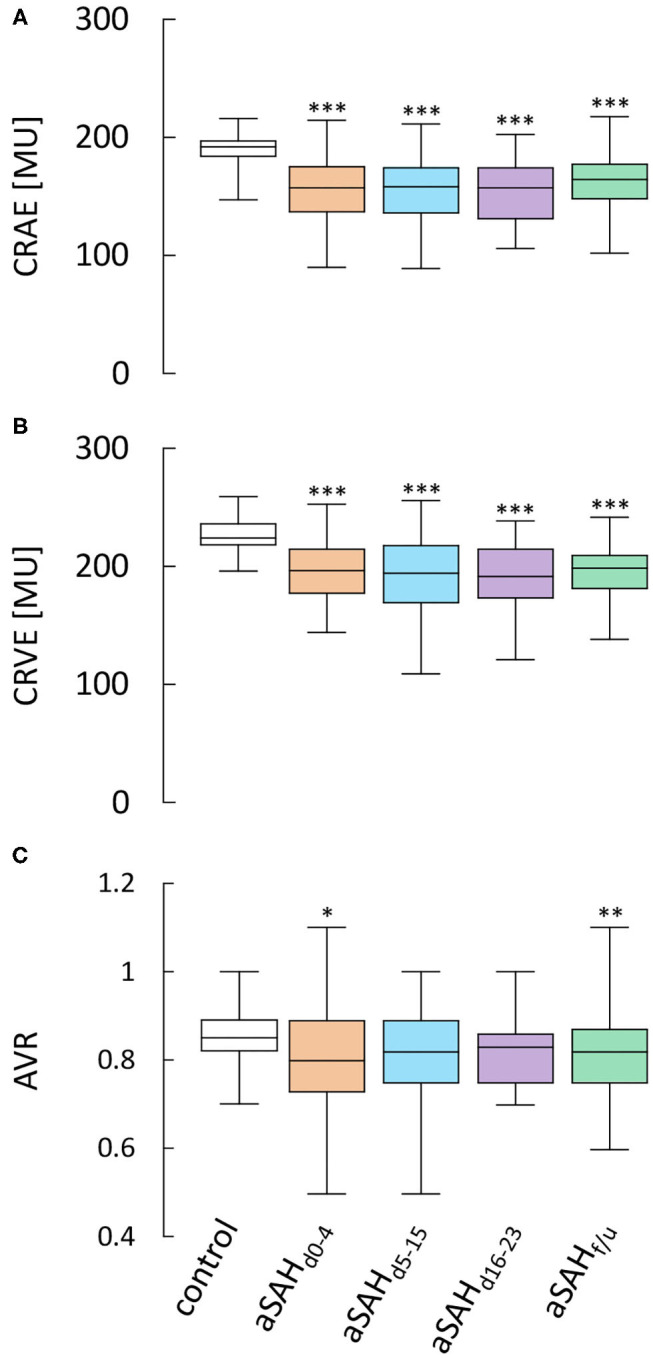
Results from static RVA in aSAH patients and control subjects. Comparison of **(A)** central retinal arterial equivalent (CRAE) as a measure of arterial diameter, **(B)** central retinal venous equivalent (CRVE) as a measure of venous diameter and **(C)** retinal arterio-venous-ratio (AVR) in control subjects and aSAH patients during the early (aSAH_d0−4_), critical (aSAH_d5−15_) and late (aSAH_d16−23_) phase and at follow-up more than 6 weeks after the ictus (aSAH_f/u_). ***p < 0.001 vs. control; **p < 0.01 vs. control; *p < 0.05 vs. control.

When compared to control subjects, the arterial diameter, expressed as central retinal arterial equivalent (CRAE), was significantly reduced immediately after aSAH and remained so throughout the whole observation period (*p* < 0.0001 for all time periods examined), with only a minor, non-significant increase in vessel diameters observed in measurements performed at follow-up (f/u) more than 6 weeks after the ictus (Figure 2A, Table 2). Likewise, the central retinal venous equivalent (CRVE) was significantly reduced in aSAH patients compared to control subjects (*p* < 0.0001 for all time periods examined) and showed little change during the observation period (Figure 2B, Table 3). For relative assessment of vessel dimensions and to account for systematic variations in measurements, the retinal arterio-venous-ratio (AVR) was also calculated. As illustrated in Figure 2C, the AVR showed very similar changes, although the difference to the control subjects only reached statistical significance during the early phase (d_0−4_, *p* = 0.0396) and after more than 6 weeks (f/u, *p* = 0.0050).

**Table 2:** Arterial RVA parameters in control and aSAH patients.

**Parameter**	**Median [q1–q3]**	***n***	***p*-value (vs. Control)**	***p*-value (vs. aSAH_**f/u**_)**
**CRAE [MU]**				
Control	192 [184–197]	42	–	–
aSAH_d0−4_	157 [137–175]	32	** <0.0001**	0.2214
aSAH_d5−15_	158 [136–174]	53	** <0.0001**	0.3996
aSAH_d16−23_	157 [131–174]	23	** <0.0001**	0.5614
aSAH_f/u_	164 [148–177]	40	** <0.0001**	–
**AVR**				
Control	0.85 [0.82–0.89]	42	**–**	–
aSAH_d0−4_	0.80 [0.73–0.89]	32	**0.0396**	0.8626
aSAH_d5−15_	0.82 [0.75–0.89]	53	0.0802	0.4305
aSAH_d16−23_	0.83 [0.75–0.86]	23	0.0986	0.7186
aSAH_f/u_	0.82 [0.75–0.87]	40	**0.0050**	–
**MAD [%]**				
Control	3.8 [2.6–5.6]	42	–	–
aSAH_d0−4_	2.2 [1.0–3.2]	32	**0.0016**	0.0521
aSAH_d5−15_	2.1 [1.4–3.6]	53	**0.0002**	**0.0321**
aSAH_d16−23_	2.9 [1.6–4.2]	23	**0.0189**	0.2623
aSAH_f/u_	3.0 [2.0–5.0]	40	0.1410	–
**tMAD [s]**				
Control	19.0 [17.0–21.0]	42	–	–
aSAH_d0−4_	17.0 [14.0–22.0]	32	0.2638	0.6574
aSAH_d5−15_	18.0 [14.8–19.8]	53	0.1295	0.7246
aSAH_d16−23_	20.0 [17.3–21.8]	23	0.7620	0.0907
aSAH_f/u_	18.0 [12.3–20.0]	40	0.0830	–
**tMAD**_**30**_ **[s]**				
Control	5.0 [4.1–5.5]	42	–	–
aSAH_d0−4_	5.0 [4.0–8.0]	32	0.1762	**0.0266**
aSAH_d5−15_	5.0 [3.0–7.0]	53	0.8656	0.1599
aSAH_d16−23_	5.0 [3.0–6.0]	23	0.8524	0.3451
aSAH_f/u_	4.0 [2.5–5.5]	40	0.0680	–
**AUC**_**art**_ [%*s]				
Control	51.4 [32.5–69.7]	42	–	–
aSAH_d0−4_	21.5 [9.4–35.8]	32	**0.0001**	**0.0066**
aSAH_d5−15_	22.4 [12.6–45.0]	53	** <0.0001**	**0.0047**
aSAH_d16−23_	30.5 [3.2–53.9]	23	**0.0033**	0.0890
aSAH_f/u_	44.5 [23.2–61.1]	40	0.1380	–

*AUC_art_, arterial area under the curve during flicker stimulation; AVR, retinal arterio-venous-ratio; CRAE, central retinal arterial equivalent; f/u, follow-up (>6 weeks after ictus); MAD, maximum arterial dilation; RVA, retinal vessel analysis; tMAD, time to maximum arterial dilation; tMAD_30_, time to 30% of maximum arterial dilation. Bold values indicate p <0.05*.

**Table 3:** Venous RVA parameters in control and aSAH patients.

**Parameter**	**Median [q1–q3]**	***n***	***p*-value (vs. Control)**	***p*-value (vs. aSAH_**f/u**_)**
**CRVE [MU]**				
Control	224 [218–236]	42	–	–
aSAH_d0−4_	196 [177–214]	32	** <0.0001**	0.2909
aSAH_d5−15_	194 [169–217]	53	** <0.0001**	0.4043
aSAH_d16−23_	191 [173–214]	23	** <0.0001**	0.2454
aSAH_f/u_	198 [181–209]	40	** <0.0001**	–
**AVR**				
Control	0.85 [0.82–0.89]	42	**–**	–
aSAH_d0−4_	0.80 [0.73–0.89]	32	**0.0396**	0.8626
aSAH_d5−15_	0.82 [0.75–0.89]	53	0.0802	0.4305
aSAH_d16−23_	0.83 [0.75–0.86]	23	0.0986	0.7186
aSAH_f/u_	0.82 [0.75–0.87]	40	**0.0050**	–
**MVD [%]**				
Control	3.9 [3.0–5.8]	42	–	–
aSAH_d0−4_	3.7 [2.9–4.7]	32	0.3179	0.1432
aSAH_d5−15_	4.1 [2.7–5.2]	53	0.4165	0.2388
aSAH_d16−23_	4.3 [2.6–5.4]	23	0.6356	0.5304
aSAH_f/u_	4.5 [3.4–5.4]	40	0.7270	–
**tMVD [s]**				
Control	20.5 [19.0–22.0]	42	–	–
aSAH_d0−4_	22.0 [20.0–24.0]	32	0.1872	**0.0235**
aSAH_d5−15_	21.0 [19.0–22.5]	53	0.9903	0.0761
aSAH_d16−23_	22.0 [20.0–24.0]	23	0.4663	**0.0110**
aSAH_f/u_	19.0 [18.0–22.0]	40	0.2040	–
**tMVD**_**30**_ **[s]**				
Control	7.0 [6.0–8.4]	42	–	–
aSAH_d0−4_	6.0 [5.0–8.0]	32	0.1269	0.6026
aSAH_d5−15_	7.0 [6.0–8.0]	53	0.7015	0.3347
aSAH_d16−23_	7.0 [7.0–8.0]	23	0.0720	0.1297
aSAH_f/u_	6.5 [5.5–8.0]	40	0.1530	–
**AUC**_**ven**_ [%*s]				
Control	45.7 [31.3–62.2]	42	–	–
aSAH_d0−4_	41.3 [16.4–46.8]	32	0.0610	**0.0205**
aSAH_d5−15_	45.0 [27.5–60.8]	53	0.3833	0.1464
aSAH_d16−23_	44.5 [20.2–60.9]	23	0.3054	0.1853
aSAH_f/u_	52.4 [34.2–61.7]	40	0.6030	–

*AUC_ven_, venous area under the curve during flicker stimulation; AVR, retinal arterio-venous-ratio; CRVE, central retinal venous equivalent; f/u, follow-up (>6 weeks after ictus); MVD, maximum venous dilation; RVA, retinal vessel analysis; tMVD, time to maximum venous dilation; tMVD_30_, time to 30% of maximum venous dilation. Bold values indicate p <0.05*.

### Neurovascular Coupling

**Figure 3:**
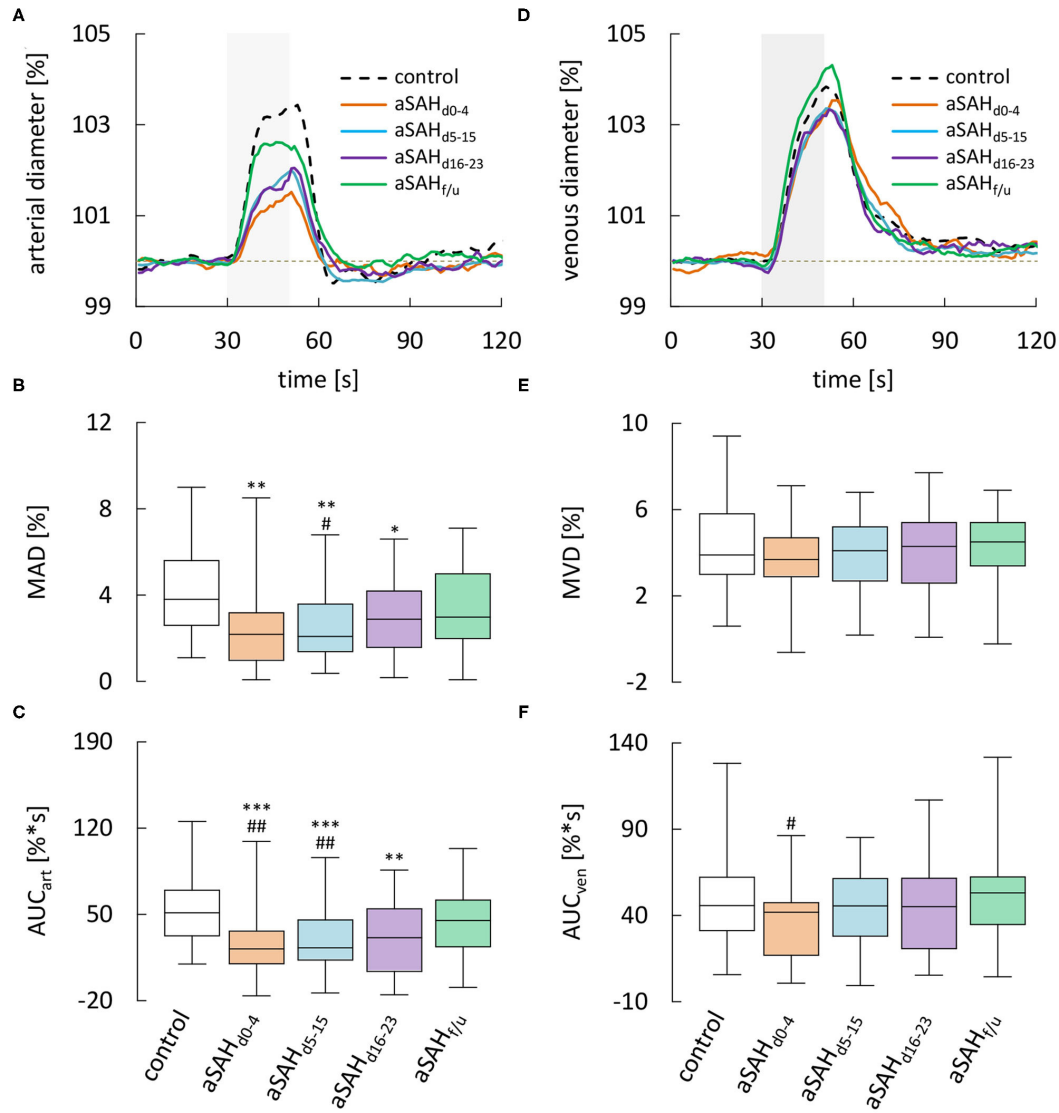
Results from dynamic RVA in aSAH patients and control subjects. **(A)** Average relative retinal arterial responses to flicker light stimulation measured in control subjects and aSAH patients during the early (aSAH_d0−4_), critical (aSAH_d5−15_), and late (aSAH_d16−23_) phase and at follow-up more than 6 weeks after the ictus (aSAH_f/u_). Stimulation period is indicated by light gray shading. **(B)** Maximum arterial dilation (MAD) quantified from data shown in A. **(C)** Arterial area under the curve (AUC_art_ ) quantified from the data shown in A. **(D)** Average retinal venous responses to flicker light stimulation measured in control subjects and aSAH patients at the same times as in A. **(E)** Maximum venous dilation (MVD) quantified from the data shown in D. **(F)** Venous area under the curve (AUC_ven_) quantified from the data shown in A. ***p < 0.001 vs. control; **p < 0.01 vs. control; *p = 0.05 vs. control; ^*##*^*p* < 0.01 vs. aSAH_f/u_; ^#^*p* < 0.05 vs. aSAH_f/u_.

Dynamic retinal vessel analysis revealed dramatic and sustained impairments in the arterial response of aSAH patients to flicker light stimulation when compared to the control subjects (Figure 3A). In particular, aSAH patients exhibited an acute reduction in arterial vasodilation during the flicker, as reflected in a significantly lower MAD [2.2 (1.0–3.2)% vs. 3.8 (2.6–5.6)% in control subjects, *p* = 0.0016] and AUC_art_ [21.5 (9.4–35.8)%^*^s vs. 51.4 (32.5–69.7)%^*^s in control subjects, *p* = 0.0001] during the early phase (d_0−4_, Figures 3B,C), while the timing of the response (i.e., tMAD and tMAD_30_) appeared to be largely unaffected (Table 2). Unlike the changes in arterial diameter and AVR, both MAD and AUC_art_ showed a gradual recovery toward healthy controls, with a significant reduction only during the first 3 weeks after the ictus (Figures 3B,C). For example, AUC_art_ increased to 22.4 [12.6–45.0]%^*^s (*p* < 0.0001 vs. Control) during the critical (d_5−15_) and 30.5 [3.2–53.9]%^*^s (*p* = 0.0033 vs. Control) during the late (d_16−23_) phase (Figure 3C). In measurements performed at follow-up more than 6 weeks after the ictus, AUC_art_ already amounted to 44.5 [23.2–61.1]%^*^s, which is close to the value of 51.4 [32.5–69.7]%^*^s (*p* = 0.1380) observed in the control subjects and significantly higher than the values observed during the early [21.5 (9.4–35.8)%^*^s, *p* = 0.0066] and critical [22.4 (12.6–45.0)%^*^s, *p* = 0.0047] phase (Figure 3C).

Analysis of venous responses to flicker light revealed much less marked differences (Figure 3D, Table 3). In particular, neither MVD (Figure 3E) nor the timing of venous responses (Table 3) showed significant differences compared to control subjects. Moreover, there was only a tendency for AUC_ven_ during the early phase to be reduced when compared to control subjects [41.3 (16.4–46.8)%^*^s vs. 45.7 (31.2–62.2)%^*^s in control subjects, *p* = 0.0610], although a significant difference was detected when compared to the corresponding value at follow-up [41.3 (16.4–46.8)%^*^s vs. 52.4 (34.2–61.7)%^*^s at follow-up, *p* = 0.0205; Figure 3F].

### Subgroup Analyses With Regard to Cardiovascular Risk Factors

Cardiovascular risk factors like diabetes mellitus have been shown to alter retinal vessel diameters and/or responses to stimulation (32) and could thus act as important confounding factors. There was only one patient with diabetes in our study, excluding further analysis of its potential as a confounding factor. However, to address the role of other cardiovascular risk factors like arterial hypertension, obesity and smoking, we examined their potential association with RVA parameters determined during the early phase. There was no difference in any of the parameters between patients stratified according to smoking status, but a tendency for AUC_ven_ to be lower in patients in hypertension [36.1 (12.8–43.2)%^*^s vs. 44.4 (27.6–56.5)%^*^s in normotensive patients, *p* = 0.0818], which may have contributed to the difference in AUC_ven_ between aSAH and control patients during the early phase described above. In addition, while AUC_art_ tended to be lower in patients with BMI ≥ 30 [8.1 (−10.1–12.5)%^*^s vs. 26.9 (11.6–36.6)%^*^s in patients with BMI <30, *p* = 0.062], it actually tended to be higher in patients with arterial hypertension [27.8 (14.9–44.8)%^*^s vs. 11.9 (4.1–32.0)%^*^s in normotensive patients, *p* = 0.0502], although none of these differences reached statistical significance (for details see Supplementary Table 1).

### Subgroup Analyses With Regard to DCI and Long-Term Clinical Outcome

**Figure 4:**
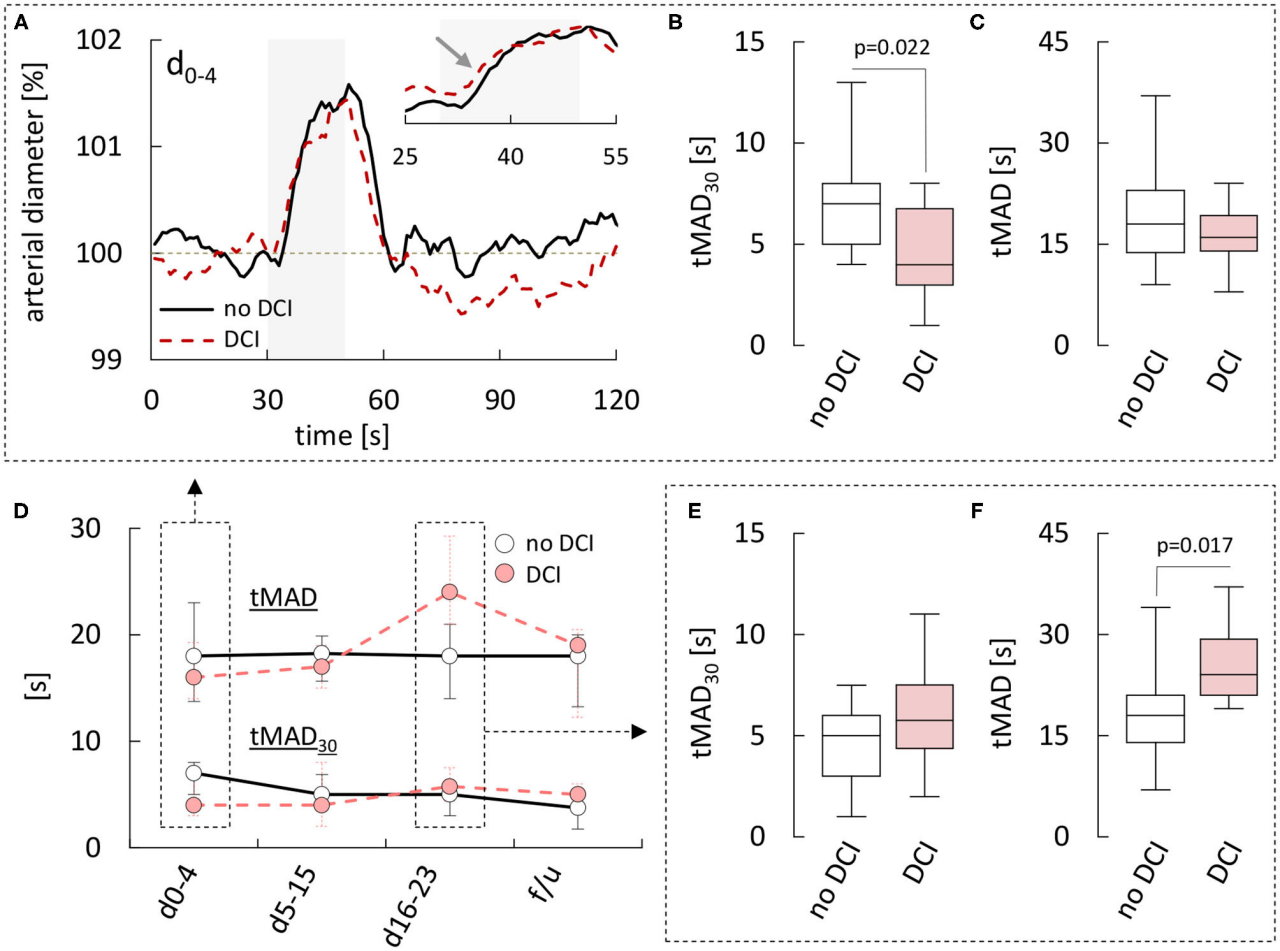
Comparison of retinal arterial responses in aSAH patients with and without DCI. **(A)** Average relative retinal arterial responses to flicker light stimulation measured during the early phase (days 0–4) in aSAH patients with or without delayed cerebral ischemia (DCI). Stimulation period is indicated by light gray shading. Inset shows the rising phase on an expanded time-scale and after scaling the responses to the same amplitude to highlight kinetic changes. **(B)** Time to 30% of the maximum arterial dilation (tMAD_30_) and **(C)** time to maximum arterial dilation (tMAD) determined during the early phase in patients with and without DCI. **(D)** Time-course of changes in median [q1–q3] tMAD_30_ (bottom) and tMAD (top) observed in patients with or without DCI. **(E)** tMAD_30_ and **(F)** tMAD determined during the late phase (days 16–23) in patients with and without DCI. For a summary of all parameters and *p*-values see Supplementary Table 2.

To examine if differences in retinal vessel responses could facilitate early detection of patients at risk of complications and delayed brain injury, we also analyzed data stratified according to the occurrence of DCI and long-term clinical outcome. While most parameters were independent of the subgroup (Supplementary Tables 2, 3), there were some subtle but significant kinetic differences between arterial responses in patients with and without DCI. In particular, patients that developed DCI showed faster vasodilation on flicker initiation during the early phase (Figure 4A), which was most evident when the average responses in both groups were normalized by their maximum amplitude (inset and arrow in Figure 4A). Quantitatively, this difference was reflected in a significantly lower tMAD_30_ (Figure 4B), which amounted to 4.0 [3.0–6.8] s in patients with DCI (*n* = 10) vs. 7.0 [5.0–8.0] s (*p* = 0.022) in patients without DCI (*n* = 13). There was also a tendency for a lower tMAD in patients with DCI, but the difference was much less pronounced and did not reach statistical significance (*p* = 0.254, Figure 4C). Interestingly, these differences appeared to be restricted to the early phase and their direction actually reversed during the late phase (Figure 4D), where patients with DCI exhibited a higher tMAD_30_ (Figure 4E) and a significantly higher tMAD [24.0 (21.0–29.3) s, *n* = 6 vs. 18.0 (14.0–21.0) s in patients without DCI, *n* = 17, *p* = 0.017, Figure 4F]. In contrast, no significant differences were observed in venous retinal vessel parameters between patients with and without DCI (Supplementary Table 2). Comparison of results with regard to the clinical outcome after 12 months was hampered by the fact that RVA could only be performed in a total of six patients with a poor clinical outcome. However, patients with an unfavorable outcome were characterized by a tendency for lower arterial and venous diameters throughout the whole observation period (Supplementary Table 3), and the CRVE in these patients during the critical phase [d_5−15_: 171 (144–187) MU, *n* = 4] was significantly (*p* = 0.045) lower than the corresponding value in patients with a favorable outcome [199 (170–220) MU, *n* = 43]. Arterial and venous parameters from dynamic retinal vessel analysis on the other hand either showed no significant differences or the number of patients with a poor outcome was simply too small for statistical testing (Supplementary Table 3).

### Effects of Nimodipine Treatment

**Figure 5:**
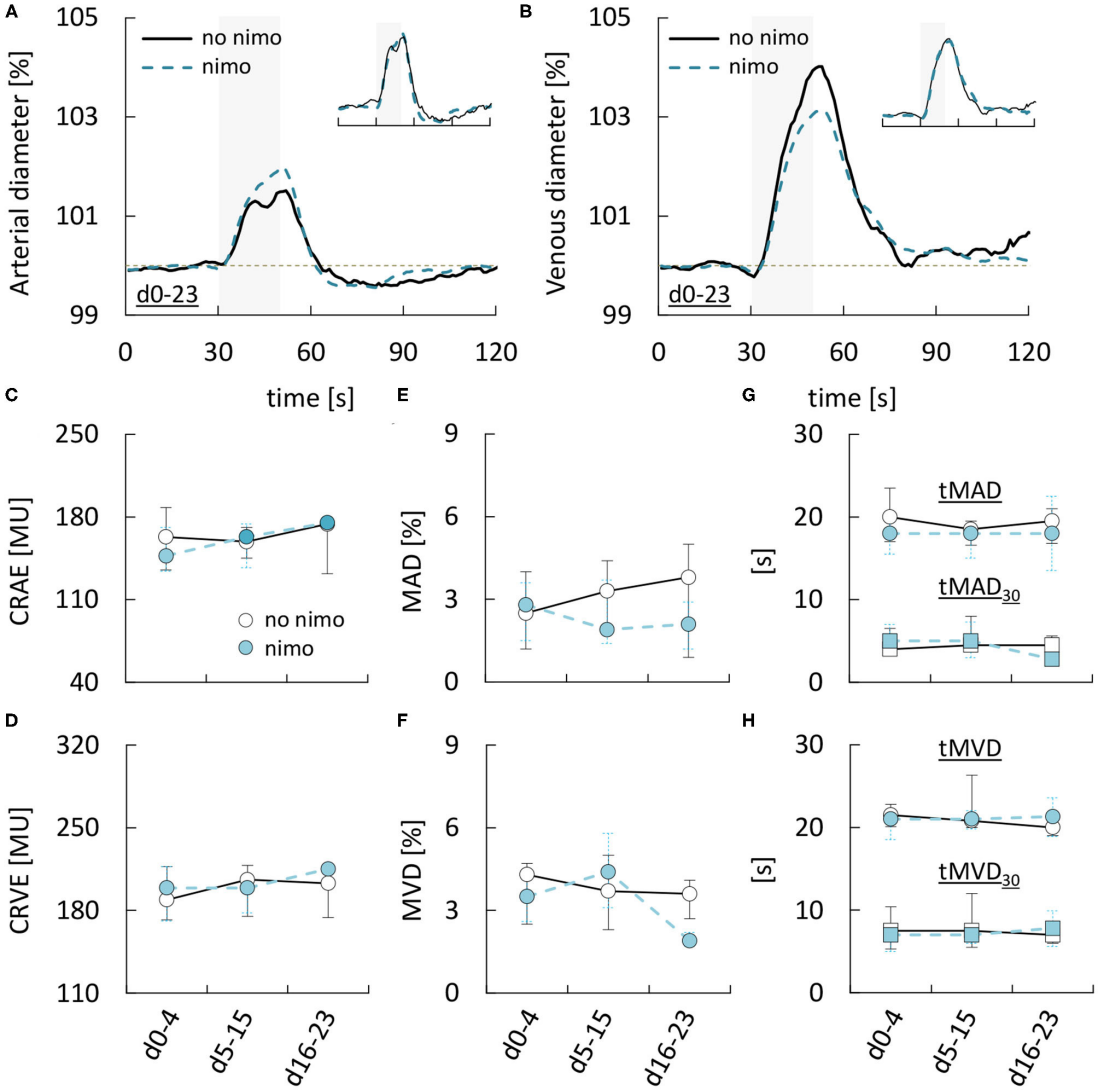
Dependence of retinal vessel properties in aSAH patients on nimodipine treatment. **(A)** Average relative arterial responses to flicker light stimulation measured with or without nimodipine (nimo) treatment. Inset shows the same data but scaled to the same amplitude for comparison of response kinetics. Stimulation period is indicated by light gray shading. **(B)** Average relative venous responses to flicker light stimulation measured with or without nimodipine (nimo) treatment. Inset shows the same data but scaled to the same amplitude for comparison of response kinetics. Stimulation period is indicated by light gray shading. **(C–H)** Comparison of **(C)** central retinal arterial equivalent (CRAE), **(D)** central retinal venous equivalent (CRVE), **(E)** maximum arterial dilation (MAD), **(F)** maximum venous dilation (MVD), **(G)** time to 30 and 100% of maximum arterial dilation (tMAD_30_ & tMAD), and **(H)** time to 30 and 100% of maximum venous dilation (tMVD_30_ & tMVD) in aSAH patients with or without nimodipine treatment during the early (aSAH_d0−4_), critical (aSAH_d5−15_), and late (aSAH_d16−23_) phase.

Finally, to examine if retinal vessel diameters or their response to flicker stimulation were affected by treatment with the L-type Ca^2+^ channel antagonist nimodipine, we compared data obtained during nimodipine treatment and data obtained while no nimodipine was administered (Supplementary Table 4). However, neither static nor dynamic retinal vessel parameters appeared to be affected by nimodipine. For example, in 51 static measurements performed during nimodipine treatment, arterial and venous diameters (CRAE & CRVE) were 158 [136–174] MU and 199 [171–214] MU, while the corresponding values in 30 measurements performed while no nimodipine was administered were 162 [133–178] MU (*p* = 0.4868) and 200 [174–209] MU (*p* = 0.9692). Likewise, maximum arterial and venous dilation upon stimulation (MAD and MVD) amounted to 2.2 [1.4–3.6]% and 3.6 [2.6–5.0]% during nimodipine treatment as compared to 2.9 [1.2–4.6]% (*p* = 0.4343) and 3.7 [2.4–4.7]% (*p* = 0.8484) in measurements performed while no nimodipine was administered (Figures 5A,B, for remaining parameters see Supplementary Table 4). Similar results were obtained when the same analysis was performed after stratification of the patients according to the occurrence of DCI (see Supplementary Table 4). Finally, to ensure that this was not due to differences in the time-frame of measurements performed with and without nimodipine treatment, we also compared data obtained during early, critical and late phase respectively (Figures 5C–H, Supplementary Table 6). However, neither retinal vessel diameters (Figures 5C,D), nor the maximum amplitude (Figures 5E,F), AUC (Supplementary Table 6), or time-course (Figures 5G,H) of vasodilation in response to flicker light stimulation during any of the phases appeared to be affected by nimodipine treatment.

## Discussion

The exact pathophysiological cascades underlying delayed cerebral ischemia (DCI) and other complications in aSAH patients surviving the initial bleed are still poorly understood. Evidence from animal models indicates that a mismatch between metabolic demand and local CBF due to impaired NVC could contribute to DCI, but the clinical relevance of these findings has remained elusive as there is still a lack of methods for non-invasive assessment of NVC in human subjects. A promising approach to evaluate NVC in aSAH patients is to measure microvascular responses in the retina, an embryological part of the central nervous system and possibly exposed to the same vasoactive hemoglobin metabolites and inflammatory cytokines as small cerebral vessels. Thus, recent discovery of a cerebral and retinal glymphatic system (22) indicates that there are perivascular pathways through which vasoactive agents formed in the subarachnoid space could directly reach the retinal microvasculature. Here, we used static and dynamic retinal vessel analysis (RVA) in aSAH patients to detect changes in basal diameter and responsiveness of retinal vessels to flicker light stimulation, both of which have been proposed as potential candidates for early detection of cerebrovascular dysfunction (14, 15).

### Static RVA and Retinal Vessel Diameters After aSAH

Vasoconstriction of large caliber proximal vessels (i.e., angiographic vasospasm) is a common complication after aSAH and was long regarded as the main cause of DCI and poor clinical outcomes, but more recent studies have questioned this concept and proposed a more important role of microvascular changes (4, 5). Our present findings point to a sustained reduction in the diameter of retinal microvessels after aSAH compared to control subjects, which appeared to be more pronounced in the arterial compartment, as reflected in a parallel and significant decrease of the arterio-venous-ratio (AVR) during the early phase and at follow-up. The latter would be consistent with the general view that veins have only limited capability for active vasoconstriction and our previous proposal that the reduced venule diameter in the retina after aSAH is at least partly mediated by constriction of upstream arterioles and a resulting reduction in blood flow (11). In any case, the observed microvascular changes resemble aSAH-induced changes in large caliber vessels, namely early cerebral artery vasoconstriction and an associated reduction in cerebral perfusion which may contribute to DCI in some patients (33–35). Considering that increased intracranial pressure during the acute phase of aSAH forces subarachnoid blood into the preretinal space, a significant reduction in retinal vessel diameters observed during the early phase after aSAH is in line with previous *in vitro* and *in vivo* animal studies showing that blood-filled cerebrospinal fluid enhances constriction of both, the retinal (36) and cerebral (37) microvasculature. Interestingly and in contrast to angiographic vasospasm, which usually dissolves by week 2 after the ictus (34), however, there was almost no return of arterial and venous diameters toward the control level during the first 3 weeks after aSAH and only a partial, non-significant recovery at follow-up more than 6 weeks later. A possible explanation for these observations is that part of the reduced basal vessel diameter reflects pathophysiological mechanisms that are not readily reversible and outlast the impairments in vessel responsiveness to stimulation. This would be in line with the results from animal studies indicating that, at least in large caliber vessels, the initial arterial narrowing by vasoactive substances after aSAH leads to tissue damage and structural changes with an associated long-term alteration of the arterial tone (38, 39). In this context, it is also interesting to note that perivascular enlargement in the brain, a putative imaging biomarker for microvascular brain damage (40), has recently been demonstrated to be associated with narrower arterial calibers in the retina (41).

### Dynamic RVA and Neurovascular Coupling After aSAH

Adequate NVC is critical for continuous adjustment of local CBF to regionally heterogeneous changes in metabolic demand but may be compromised by the pathophysiological cascades initiated during bleeding into the subarachnoid space. Our present findings demonstrate a sustained decrease of retinal arterial vasodilation with partial recovery in response to flicker light stimulation in aSAH patients, as reflected in a significantly reduced maximum amplitude and area under the response curve compared to control subjects. Unlike the changes in vessel diameters, arterial responsiveness in aSAH patients showed a gradual recovery during the first 3 weeks and reached almost normal levels at follow-up more than 6 weeks after the ictus. Given that complications like DCI and DCI-related infarctions, which have been proposed to be the result of microvascular dysfunction in the brain, are typically restricted to the first two or at most 3 weeks after the ictus as well, these findings support the assumption that retinal vessels could serve as a surrogate marker for changes in cerebral small vessels after aSAH. The observed time-course also matches the pathophysiological cascades traditionally thought to be responsible for vascular dysfunction after aSAH, namely hemolysis of subarachnoid blood and intrathecal immune activation during the early phase, secondary immune infiltration and formation of various vasoactive hemoglobin metabolites during the critical phase and their gradual clearance during the late phase (42–44). Further support for the idea comes from recent discovery of cerebral and retinal glymphatic systems (22), which could provide a perivascular pathway through which vasoactive agents formed during the critical phase reach the retinal microvasculature. If this turns out to be the case, dynamic RVA could not only provide a unique tool to monitor their impact on microvascular function in a relatively simple manner but also a read-out of disease- or treatment-related changes in cerebral small vessels that are otherwise difficult or impossible to assess directly and non-invasively. For example, our present findings suggest that nimodipine, the only FDA-approved pharmacological treatment for delayed cerebral vasospasm after aSAH (45), neither affects the basal diameter nor the responsiveness of retinal microvessels to stimulation, although this remains to be confirmed it future studies.

Likewise, further study will be required to evaluate the sensitivity of RVA with regard to the early detection of DCI.

### Limitations and Confounding Factors

There are several confounding factors that have to be taken into account when interpreting our results. First and foremost, although adaptation of our current imaging technique allowed us to also recruit some more severely affected, analgo-sedated patients, cases with mild aSAH, good clinical grade (HH_1−3_), and/or good clinical outcome were still clearly overrepresented in our cohort, which may have biased the observed changes, likely toward milder alterations. To overcome this limitation, which is mainly brought about by the limited maneuverability of the required funduscope in supine patients, we are currently working on a miniaturized, wearable device for RVA that is comparable to commercially available “smartglasses” and should facilitate bedside testing of immobilized and sedated patients (46). That said, given the complexity of blood flow regulation and NVC, it is likely that numerous factors, some of which may still be unknown, can influence retinal vessel diameters and their responsiveness to stimulation. In order to reduce potential confounding factors and ensure standardized, physiological conditions at the time of image acquisition, our measurements were routinely paralleled by multimodal monitoring of mean arterial blood pressure (MAP), heart rate, blood gases, oxygenation, and intracranial pressure (ICP). In addition, our analysis indicates that there was no effect of nimodipine treatment on any of the parameters evaluated. With regard to static RVA, it is also important to keep in mind that direct comparison of CRAE and CRVE values between control and aSAH patients may have been influenced by the fact that measurements were performed using devices at two different facilities.

It is also worth noting that cardiovascular risk factors like diabetes or arterial hypertension could act as confounding factors and complicate the use of RVA in the context of aSAH. For example, in the present study, there was a tendency for AUC_art_ to be higher in patients with arterial hypertension, which could partly mask the decrease of this parameter after aSAH. Consistent with previous studies (32), these differences were relatively subtle and they did not reach statistical significance, but further studies on the role of these and other cardiovascular risk factors will clearly be required before RVA can be reliably used for diagnostic applications in the context of aSAH.

Finally, it should be noted that in contrast to our previous study, we observed no significant effects of aSAH on the latency of arterial or venous responses, which could reflect the fact that these parameters are more sensitive to inherent technical limitations such as the finite signal-to-noise ratio. As such, our present findings that patients with DCI exhibit a reduced arterial latency (tMAD_30_) during the early but increased arterial latency (tMAD) during the late phase should be interpreted with care as well-until they can be confirmed in future studies.

In particular, given the subtle nature of these differences, it seems unlikely that they would allow for reliable prediction and/or confirmation of DCI in the clinical setting. However, based on the small number of patients we evaluated, future studies with larger patient populations may be able to identify more robust correlations between RVA parameters and DCI. Considering that DCI remains one of the main preventable causes of poor clinical outcomes after aSAH, such studies will almost certainly be performed in the near future and their results should help to conclusively rate the clinical value of RVA in this setting.

## Conclusions

Our findings confirm and extend the results from previous studies that aSAH is associated with sustained vasoconstriction and impairments of NVC in retinal vessels. Retinal vessel responses may differ between patients with and without DCI. Although their clinical relevance remains to be firmly established, these findings suggest that RVA could be a unique tool for monitoring microvascular function in aSAH patients in a simple and non-invasive manner.

## Data Availability Statement

The original contributions presented in the study are included in the article/Supplementary Material, further inquiries can be directed to the corresponding author/s.

## Ethics Statement

All procedures involving human participants were performed in accordance with the ethical standards of the institutional and/or national research committee and with the 1964 Helsinki declaration and its later amendments or comparable ethical standards. Reference Number EK 069/15. The patients/participants provided their written informed consent to participate in this study.

## Author Contributions

WA, CC, MWe, KS, DK, and GS: conceived, designed, and performed the experiments. WA: first drafting of the manuscript. WA and FN: illustrations. WA, CC, MWe, KS, KK, and GS: data acquisition. WA, CC, KK, and GS: analysis and interpretation of data. WA, CC, TS, JH, AB, AS-T, FN, MWi, HC, and GS: critical review of the manuscript. All authors were critically revised and approved by the final manuscript.

## Conflict of Interest

IMEDOS Systems GmbH provided the Retinal Vessel Analyzer for research purposes only. The company did not have any additional role in the study design, data collection and analysis, decision to publish, or preparation of the manuscript. The authors declare that the research was conducted in the absence of any commercial or financial relationships that could be construed as a potential conflict of interest.
