# Supplementary material for: Non-invasive Assessment of Neurovascular Coupling After Aneurysmal Subarachnoid Hemorrhage: A Prospective Observational Trial Using Retinal Vessel Analysis
Source: Front Neurol. 2021 Jun 14;12:690183. doi: 10.3389/fneur.2021.690183 (PMC8236540; doi:10.3389/fneur.2021.690183)
Supplement: Supplementary file 1 [file Data_Sheet_1.docx]

Suppl. Table 1. RVA parameters in aSAH patients with different cardiovascular risk factors.

| **Parameter** | **normotension** | |  | **hypertension** | | **p-value** |
| --- | --- | --- | --- | --- | --- | --- |
|  | median [q1-q3] |  |  | median [q1-q3] |  |  |
| **n** | 16 |  |  | 16 |  |  |
| **CRAE [MU]** | 162 [140-182] |  |  | 157 [130-165] |  | 0.1291 |
| **CRVE [MU]** | 207 [182-224] |  |  | 185 [176-201] |  | 0.1142 |
| **AVR** | 0.8 [0.7-0.9] |  |  | 0.8 [0.7-0.9] |  | 0.9007 |
| **MAD [%]** | 1.3 [0.7-2.9] |  |  | 2.5 [1.8-4.7] |  | 0.0681 |
| **tMAD [s]** | 17.5 [14.0-22.5] |  |  | 17.0 [12.0-20.0] |  | 0.5485 |
| **tMAD_30_ [s]** | 5.5 [4.3-7.8] |  |  | 5.0 [4.0-8.0] |  | 0.6610 |
| **AUC_art_ [%*s]** | 11.9 [4.1-32.0] |  |  | 27.8 [14.9-44.8] |  | 0.0502 |
| **MVD [%]** | 4.2 [3.1-5.5] |  |  | 3.3 [2.6-4.2] |  | 0.2106 |
| **tMVD [s]** | 22.0 [19.0-22.0] |  |  | 23.0 [20.0-25.5] |  | 0.2622 |
| **tMVD_30_ [s]** | 6.5 [5.0-8.0] |  |  | 6.0 [5.0-8.3] |  | 0.8052 |
| **AUC_ven_ [%*s]** | 44.4 [27.6-56.5] |  |  | 36.1 [12.8-43.2] |  | 0.0818 |

| **Parameter** | **BMI<30** | |  | **BMI≥30** | | **p-value** |
| --- | --- | --- | --- | --- | --- | --- |
|  | median [q1-q3] |  |  | median [q1-q3] |  |  |
| **n** | 25 |  |  | 7 |  |  |
| **CRAE [MU]** | 160 [140-175] |  |  | 129 [121-169] |  | 0.3000 |
| **CRVE [MU]** | 192 [176-220] |  |  | 202 [186-206] |  | 0.9170 |
| **AVR** | 0.8 [0.7-0.9] |  |  | 0.8 [0.6-0.8] |  | 0.3640 |
| **MAD [%]** | 2.4 [1.2-3.4] |  |  | 0.8 [0.5-2.2] |  | 0.1350 |
| **tMAD [s]** | 17.5 [14.4-20.5] |  |  | 13.0 [12.0-37.0] |  | 0.9460 |
| **tMAD_30_ [s]** | 5.0 [4.0-7.0] |  |  | 9.5 [6.3-11.3] |  | 0.2190 |
| **AUC_art_ [%*s]** | **26.9 [11.6-36.6]** |  |  | **8.1 [-10.1-12.5]** |  | **0.0620** |
| **MVD [%]** | 3.4 [2.9-4.6] |  |  | 5.5 [3.9-5.9] |  | 0.1440 |
| **tMVD [s]** | 21.5 [18.8-24.0] |  |  | 22.0 [22.0-23.0] |  | 0.2740 |
| **tMVD_30_ [s]** | 6.3 [5.0-8.0] |  |  | 6.0 [6.0-7.0] |  | 0.9450 |
| **AUC_ven_ [%*s]** | 37.1 [16.1-46.5] |  |  | 46.8 [44.4-47.7] |  | 0.2770 |

| **Parameter** | **non-smoker** | |  | **smoker** | | **p-value** |
| --- | --- | --- | --- | --- | --- | --- |
|  | median [q1-q3] |  |  | median [q1-q3] |  |  |
| **n** | 22 |  |  | 10 |  |  |
| **CRAE [MU]** | 160 [138-175] |  |  | 151 [123-172] |  | 0.5530 |
| **CRVE [MU]** | 188 [172-216] |  |  | 201 [186-206] |  | 0.6280 |
| **AVR** | 0.8 [0.8-0.9] |  |  | 0.8 [0.6-0.8] |  | 0.1180 |
| **MAD [%]** | 2.5 [1.0-4.0] |  |  | 1.5 [0.9-2.0] |  | 0.2370 |
| **tMAD [s]** | 17.0 [14.0-20.0] |  |  | 22.0 [15.5-28.5] |  | 0.1820 |
| **tMAD_30_ [s]** | 5.0 [4.0-7.3] |  |  | 7.0 [5.0-8.0] |  | 0.4380 |
| **AUC_art_ [%*s]** | 29.9 [11.4-35.8] |  |  | 12.5 [3.3-14.7] |  | 0.1640 |
| **MVD [%]** | 3.7 [2.9-5.0] |  |  | 3.7 [2.6-4.7] |  | 0.9300 |
| **tMVD [s]** | 21.0 [18.0-23.0] |  |  | 22.5 [21.8-24.3] |  | 0.1350 |
| **tMVD_30_ [s]** | 6.5 [6.0-8.0] |  |  | 5.5 [4.3-8.0] |  | 0.5380 |
| **AUC_ven_ [%*s]** | 38.0 [24.1-47.7] |  |  | 45.4 [16.1-46.7] |  | 0.9070 |

**Suppl. Table 2. RVA parameters in aSAH patients with and without DCI.**

| **Parameter** | **no DCI** | |  | **DCI** | | **p-value** |
| --- | --- | --- | --- | --- | --- | --- |
|  | median [q1-q3] | n |  | median [q1-q3] | n |  |
|  |  |  |  |  |  |  |
| **CRAE [MU]** |  |  |  |  |  |  |
| Day 0-4 | 157 [136-175] | 20 |  | 160 [139-175] | 10 | 0.982 |
| Day 5-15 | 156 [140-175] | 36 |  | 158 [131-172] | 11 | 0.973 |
| Day 16-23 | 167 [128-174] | 13 |  | 140 [132-152] | 4 | 0.422 |
| Follow-up (>6 weeks) | 161 [150-170] | 18 |  | 171 [144-183] | 12 | 0.628 |
| **CRVE [MU]** |  |  |  |  |  |  |
| Day 0-4 | 197 [176-214] | 20 |  | 194 [185-212] | 10 | 1.000 |
| Day 5-15 | 194 [167-220] | 36 |  | 194 [174-210] | 11 | 0.633 |
| Day 16-23 | 189 [174-218] | 13 |  | 180 [156-195] | 4 | 0.156 |
| Follow-up (>6 weeks) | 202 [183-208] | 18 |  | 190 [178-216] | 12 | 0.856 |
| **AVR** |  |  |  |  |  |  |
| Day 0-4 | 0.80 [0.73-0.86] | 20 |  | 0.81 [0.67-0.92] | 10 | 0.930 |
| Day 5-15 | 0.81 [0.75-0.88] | 36 |  | 0.84 [0.81-0.92] | 11 | 0.334 |
| Day 16-23 | 0.77 [0.75-0.84] | 13 |  | 0.86 [0.82-0.90] | 4 | 0.255 |
| Follow-up (>6 weeks) | 0.82 [0.74-0.87] | 18 |  | 0.81 [0.78-0.87] | 12 | 0.739 |
| **MAD [%]** |  |  |  |  |  |  |
| Day 0-4 | 2.2 [1.1-3.1] | 15 |  | 2.2 [0.9-4.9] | 10 | 0.739 |
| Day 5-15 | 2.6 [1.6-3.8] | 34 |  | 1.9 [1.4-2.1] | 13 | 0.125 |
| Day 16-23 | 2.6 [1.5-4.3] | 17 |  | 2.7 [1.9-3.6] | 6 | 0.972 |
| Follow-up (>6 weeks) | 3.0 [2.0-4.4] | 24 |  | 4.9 [2.1-5.5] | 11 | 0.546 |
| **tMAD [s]** |  |  |  |  |  |  |
| Day 0-4 | 18.0 [13.8-23.0] | 15 |  | 16.0 [14.0-19.3] | 10 | 0.254 |
| Day 5-15 | 18.3 [15.6-19.9] | 34 |  | 17.0 [15.0-19.0] | 13 | 0.812 |
| Day 16-23 | 18.0 [14.0-21.0] | 17 |  | 24.0 [21.0-29.3] | 6 | **0.017** |
| Follow-up (>6 weeks) | 18.0 [13.3-20.0] | 24 |  | 19.0 [12.3-20.5] | 11 | 0.643 |
| **tMAD_30_ [s]** |  |  |  |  |  |  |
| Day 0-4* | 7.0 [5.0-8.0] | 13 |  | 4.0 [3.0-6.8] | 10 | **0.022** |
| Day 5-15 | 5.0 [4.0-6.9] | 34 |  | 4.0 [2.0-8.0] | 13 | 0.616 |
| Day 16-23 | 5.0 [3.0-6.0] | 17 |  | 5.8 [4.4-7.5] | 6 | 0.230 |
| Follow-up (>6 weeks) | 3.8 [1.8-5.0] | 24 |  | 5.0 [4.0-6.0] | 11 | 0.123 |
| **AUC_art_ [%*s]** |  |  |  |  |  |  |
| Day 0-4 | 14.9 [5.0-32.5] | 15 |  | 24.7 [9.9-50.8] | 10 | 0.390 |
| Day 5-15 | 28.9 [14.1-51.5] | 34 |  | 16.0 [7.9-23.8] | 13 | 0.164 |
| Day 16-23 | 28.4 [11.1-54.1] | 17 |  | 24.7 [3.3-34.6] | 6 | 0.484 |
| Follow-up (>6 weeks) | 40.9 [24.6-55.9] | 24 |  | 53.2 [18.9-74.1] | 11 | 0.804 |
| **MVD [%]** |  |  |  |  |  |  |
| Day 0-4 | 3.7 [3.0-5.1] | 16 |  | 3.7 [1.4-4.5] | 9 | 0.321 |
| Day 5-15 | 4.3 [2.9-5.3] | 36 |  | 3.6 [2.6-4.2] | 13 | 0.213 |
| Day 16-23 | 4.3 [2.6-5.3] | 17 |  | 2.9 [2.5-7.5] | 5 | 0.969 |
| Follow-up (>6 weeks) | 4.9 [3.4-5.6] | 24 |  | 4.1 [1.9-5.1] | 13 | 0.245 |
| **tMVD [s]** |  |  |  |  |  |  |
| Day 0-4 | 22.0 [20.8-24.3] | 16 |  | 20.0 [18.0-23.0] | 9 | 0.378 |
| Day 5-15 | 20.3 [19.0-22.0] | 36 |  | 21.0 [20.0-24.0] | 13 | 0.306 |
| Day 16-23 | 22.0 [20.0-24.0] | 17 |  | 22.0 [20.5-22.0] | 5 | 0.783 |
| Follow-up (>6 weeks) | 19.0 [18.0-22.0] | 24 |  | 19.0 [18.0-21.0] | 13 | 0.653 |
| **tMVD_30_ [s]** |  |  |  |  |  |  |
| Day 0-4 | 6.8 [5.8-8.1] | 16 |  | 6.0 [5.0-7.0] | 9 | 0.377 |
| Day 5-15 | 7.3 [6.0-8.0] | 36 |  | 7.0 [6.0-9.0] | 13 | 0.856 |
| Day 16-23 | 7.0 [7.0-8.0] | 17 |  | 8.0 [7.0-9.0] | 5 | 0.420 |
| Follow-up (>6 weeks) | 7.0 [5.9-8.0] | 24 |  | 6.0 [5.0-8.0] | 13 | 0.847 |
| **AUC_ven_ [%*s]** |  |  |  |  |  |  |
| Day 0-4 | 43.3 [26.7-47.8] | 16 |  | 34.2 [10.5-42.7] | 9 | 0.141 |
| Day 5-15 | 45.5 [28.0-62.0] | 36 |  | 42.2 [22.8-48.3] | 13 | 0.483 |
| Day 16-23 | 44.5 [24.4-52.4] | 17 |  | 18.4 [15.9-80.9] | 5 | 0.667 |
| Follow-up (>6 weeks) | 55.6 [36.6-62.0] | 24 |  | 45.8 [24.7-55.7] | 13 | 0.272 |

AUC_art_, arterial area under the curve during flicker stimulation; AUC_ven_, venous area under the curve during flicker stimulation; AVR, retinal arterio-venous-ratio; CRAE, central retinal arterial equivalent; CRVE, central retinal venous equivalent; DCI, delayed cerebral ischemia; MAD, maximum arterial dilation; MVD, maximum venous dilation; RVA, retinal vessel analysis; tMAD, time to maximum arterial dilation; tMAD_30_, time to 30% of maximum arterial dilation; tMVD, time to maximum venous dilation; tMVD_30_, time to 30% of maximum venous dilation.

*Due to technical reasons, the algorithm used for analysis could not define a tMAD_30_ in 2 of the 15 patients.

Suppl. Table 3. RVA parameters in aSAH patients with good and poor clinical outcome.

| **Parameter** | **GOS-E_5-8_** | |  | **GOS-E_1-4_** | | **p-value** |
| --- | --- | --- | --- | --- | --- | --- |
|  | median [q1-q3] | n |  | median [q1-q3] | n |  |
|  |  |  |  |  |  |  |
| **CRAE [MU]** |  |  |  |  |  |  |
| Day 0-4 | 157 [138-175] | 27 |  | 136 [113-156] | 3 | 0.254 |
| Day 5-15 | 158 [139-176] | 43 |  | 137 [102-169] | 4 | 0.252 |
| Day 16-23 | 145 [127-173] | 16 |  | 175 | 1 | - |
| Follow-up (>6 weeks) | 164 [149-176] | 28 |  | 161 [153-170] | 2 | 0.845 |
| **CRVE [MU]** |  |  |  |  |  |  |
| Day 0-4 | 200 [180-217] | 27 |  | 175 [162-181] | 3 | 0.104 |
| Day 5-15 | 199 [170-220] | 43 |  | 171 [144-187] | 4 | **0.045** |
| Day 16-23 | 184 [171-214] | 16 |  | 203 | 1 | - |
| Follow-up (>6 weeks) | 201 [181-213] | 27 |  | 183 [176-191] | 2 | 0.214 |
| **AVR** |  |  |  |  |  |  |
| Day 0-4 | 0.80 [0.73-0.88] | 27 |  | 0.78 [0.69-0.86] | 3 | 0.809 |
| Day 5-15 | 0.82 [0.75-0.89] | 43 |  | 0.85 [0.78-0.90] | 4 | 0.734 |
| Day 16-23 | 0.80 [0.75-0.85] | 16 |  | 0.86 | 1 | - |
| Follow-up (>6 weeks) | 0.81 [0.75-0.86] | 28 |  | 0.88 [0.87-0.89] | 2 | 0.116 |
| **MAD [%]** |  |  |  |  |  |  |
| Day 0-4 | 2.2 [0.9-3.1] | 23 |  | 4.1 [3.2-5.1] | 2 | 0.270 |
| Day 5-15 | 2.0 [1.4-3.7] | 44 |  | 2.1 [1.9-2.5] | 3 | 0.983 |
| Day 16-23 | 2.6 [1.6-4.2] | 22 |  | 3.1 | 1 | - |
| Follow-up (>6 weeks) | 3.0 [2.0-5.0] | 34 |  | 5.9 | 1 | - |
| **tMAD [s]** |  |  |  |  |  |  |
| Day 0-4 | 17.0 [13.5-21.0] | 23 |  | 22.0 [21.0-23.0] | 2 | 0.209 |
| Day 5-15 | 17.7 [14.9-19.5] | 44 |  | 21.0 [19.5-23.0] | 3 | 0.111 |
| Day 16-23 | 19.5 [16.2-21.7] | 22 |  | 21.0 | 1 | - |
| Follow-up (>6 weeks) | 18.0 [11.9-20.0] | 34 |  | 19.0 | 1 | - |
| **tMAD_30_ [s]** |  |  |  |  |  |  |
| Day 0-4* | 5.0 [4.0-8.0] | 21 |  | 5.5 [4.8-6.3] | 2 | 0.741 |
| Day 5-15 | 5.0 [3.0-7.0] | 44 |  | 6.0 [3.5-7.0] | 3 | 0.896 |
| Day 16-23 | 5.0 [3.0-6.0] | 22 |  | 6.0 | 1 | - |
| Follow-up (>6 weeks) | 4.0 [2.3-5.0] | 34 |  | 6.0 | 1 | - |
| **AUC_art_ [%*s]** |  |  |  |  |  |  |
| Day 0-4 | 14.9 [8.8-34.3] | 23 |  | 47.5 [34.5-60.6] | 2 | 0.270 |
| Day 5-15 | 22.2 [10.9-47.9] | 44 |  | 23.8 [19.1-27.5] | 3 | 0.983 |
| Day 16-23 | 27.8 [3.2-53.9] | 22 |  | 35.2 | 1 | - |
| Follow-up (>6 weeks) | 40.9 [22.8-58.9] | 34 |  | 72.7 | 1 | - |
| **MVD [%]** |  |  |  |  |  |  |
| Day 0-4 | 3.5 [2.8-4.8] | 23 |  | 4.2 [4.0-4.5] | 2 | 0.581 |
| Day 5-15 | 4.1 [2.8-5.3] | 46 |  | 2.6 [1.8-3.6] | 3 | 0.226 |
| Day 16-23 | 4.3 [2.6-5.4] | 21 |  | 1.1 | 1 | - |
| Follow-up (>6 weeks) | 4.6 [3.4-5.5] | 36 |  | 1.4 | 1 | - |
| **tMVD [s]** |  |  |  |  |  |  |
| Day 0-4 | 22.0 [19.5-23.5] | 23 |  | 22.0 [21.0-23.0] | 2 | 0.880 |
| Day 5-15 | 20.3 [19.0-22.0] | 46 |  | 23.0 [22.0-24.5] | 3 | 0.102 |
| Day 16-23 | 22.0 [20.0-24.0] | 21 |  | 17.5 | 1 | **-** |
| Follow-up (>6 weeks) | 19.0 [18.0-22.0] | 36 |  | 18.0 | 1 | - |
| **tMVD_30_ [s]** |  |  |  |  |  |  |
| Day 0-4 | 6.0 [5.0-8.0] | 23 |  | 6.0 [5.5-6.5] | 2 | 0.801 |
| Day 5-15 | 7.0 [6.0-8.0] | 46 |  | 8.0 [8.0-12.0] | 3 | 0.078 |
| Day 16-23 | 7.0 [7.0-8.0] | 21 |  | 8.0 | 1 | - |
| Follow-up (>6 weeks) | 6.5 [5.4-8.0] | 36 |  | 8.0 | 1 | - |
| **AUC_ven_ [%*s]** |  |  |  |  |  |  |
| Day 0-4 | 41.3 [15.8-47.3] | 23 |  | 42.3 [40.2-44.5] | 2 | 0.689 |
| Day 5-15 | 45.5 [27.2-61.5] | 46 |  | 25.6 [12.3-38.6] | 3 | 0.227 |
| Day 16-23 | 44.5 [21.0-60.9] | 21 |  | 4.9 | 1 | - |
| Follow-up (>6 weeks) | 53.2 [36.6-62.0] | 36 |  | 16.7 | 1 | - |

AUC_art_, arterial area under the curve during flicker stimulation; AUC_ven_, venous area under the curve during flicker stimulation; AVR, retinal arterio-venous-ratio; CRAE, central retinal arterial equivalent; CRVE, central retinal venous equivalent; MAD, maximum arterial dilation; MVD, maximum venous dilation; RVA, retinal vessel analysis; tMAD, time to maximum arterial dilation; tMAD_30_, time to 30% of maximum arterial dilation; tMVD, time to maximum venous dilation; tMVD_30_, time to 30% of maximum venous dilation.

*Due to technical reasons, the algorithm used for analysis could not define a tMAD_30_ in 2 of the 25 patients.

**Suppl. Table 4. Dependence of RVA parameters on treatment with nimodipine in all patients.**

| **Parameter** | **nimodipine**  median [q1-q3] |  | **no nimodipine**  median [q1-q3] | **p-value** |
| --- | --- | --- | --- | --- |
| **Number of measurements** | 51 |  | 30 |  |
| **CRAE [MU]** | 158 [136-174] |  | 162 [133-178] | 0.4868 |
| **CRVE [MU]** | 199 [171-214] |  | 200 [174-209] | 0.9692 |
| **AVR** | 0.80 [0.73-0.87] |  | 0.83 [0.77-0.91] | 0.1358 |
| **MAD [%]** | 2.2 [1.4-3.6] |  | 2.9 [1.2-4.6] | 0.4343 |
| **tMAD [s]** | 18.0 [14.0-20.0] |  | 19.0 [17.0-21.0] | 0.1450 |
| **tMAD_30_ [s]** | 5.0 [3.0-7.0] |  | 4.0 [3.3-6.0] | 0.3285 |
| **AUC_art_ [%*s]** | 22.6 [11.9-41.6] |  | 35.2 [9.9-58.4] | 0.3423 |
| **MVD [%]** | 3.6 [2.6-5.0] |  | 3.7 [2.4-4.7] | 0.8484 |
| **tMVD [s]** | 21.0 [19.0-22.0] |  | 20.5 [20.0-23.0] | 0.5803 |
| **tMVD_30_ [s]** | 7.0 [6.0-8.0] |  | 7.3 [5.4-8.1] | 0.7229 |
| **AUC_ven_ [%*s]** | 42.8 [24.6-52.6] |  | 40.4 [14.7-56.0] | 0.3961 |

AUC_art_, arterial area under the curve during flicker stimulation; AUC_ven_, venous area under the curve during flicker stimulation; AVR, retinal arterio-venous-ratio; CRAE, central retinal arterial equivalent; CRVE, central retinal venous equivalent; MAD, maximum arterial dilation; MVD, maximum venous dilation; RVA, retinal vessel analysis; tMAD, time to maximum arterial dilation; tMAD_30_, time to 30% of maximum arterial dilation; tMVD, time to maximum venous dilation; tMVD_30_, time to 30% of maximum venous dilation.

**Suppl. Table 5. Dependence of RVA parameters on treatment with nimodipine in patients stratified according to the occurrence of DCI.**

|  | **Patients without DCI** | | |  | **Patients with DCI** | |  |
| --- | --- | --- | --- | --- | --- | --- | --- |
| **Parameter** | **nimodipine**  median [q1-q3] |  | **no nimodipine**  median [q1-q3] | **p-value** | **nimodipine**  median [q1-q3] | **no nimodipine**  median [q1-q3] | **p-value** |
| **Number of measurements** | 35 |  | 19 |  | 16 | 11 |  |
| **CRAE [MU]** | 161 [136 – 174] |  | 161 [135 – 179] | 0.6253 | 145 [136 – 169] | 169 [133 – 175] | 0.6642 |
| **CRVE [MU]** | 200 [171 – 215] |  | 197 [172 – 208] | 0.9252 | 196 [181 – 213] | 203 [181 – 217] | 0.9202 |
| **AVR** | 0.80 [0.74 – 0.87] |  | 0.83 [0.76 – 0.89] | 0.3921 | 0.80 [0.70 – 0.88] | 0.83 [0.82 – 0.92] | 0.2848 |
| **MAD [%]** | 2.8 [1.7 – 3.9] |  | 3.5 [1.2 – 5.0] | 0.5084 | 1.9 [1.3 – 2.2] | 2.5 [1.5 – 4.1] | 0.4434 |
| **tMAD [s]** | 18.0 [13.5 – 20.0] |  | 18.5 [16.8 – 20.6] | 0.5284 | 17.8 [14.8 – 19.5] | 20.0 [17.0 – 23.0] | 0.1432 |
| **tMAD_30_ [s]** | 5.0 [4.0 – 7.0] |  | 4.0 [3.4 – 5.3] | 0.1128 | 4.1 [2.0 – 8.0] | 5.5 [3.5 – 7.5] | 0.9405 |
| **AUC_art_ [%*s]** | 35.8 [14.4 – 43.0] |  | 43.9 [9.1 – 58.3] | 0.3938 | 16.3 [9.0 – 25.2] | 27.8 [11.8 – 56.6] | 0.4298 |
| **MVD [%]** | 3.8 [3.0 – 5.2] |  | 3.6 [2.4 – 4.6] | 0.2933 | 3.5 [2.3 – 4.5] | 4.2 [2.9 – 6.2] | 0.3648 |
| **tMVD [s]** | 21.0 [18.3 – 22.0] |  | 21.0 [20.0 – 25.3] | 0.2961 | 21.5 [20.0 – 24.4] | 20.5 [20.0 – 22.0] | 0.5498 |
| **tMVD_30_ [s]** | 7.0 [6.0 – 8.0] |  | 7.3 [5.4 – 9.1] | 0.5295 | 7.0 [5.8 – 8.3] | 6.8 [5.4 – 8.0] | 0.8292 |
| **AUC_ven_ [%*s]** | 44.2 [29.5 – 52.6] |  | 35.2 [16.1 – 55.5] | 0.2009 | 40.0 [15.9 – 49.6] | 41.3 [13.5 – 84.5] | 0.8651 |

AUC_art_, arterial area under the curve during flicker stimulation; AUC_ven_, venous area under the curve during flicker stimulation; AVR, retinal arterio-venous-ratio; CRAE, central retinal arterial equivalent; CRVE, central retinal venous equivalent; MAD, maximum arterial dilation; MVD, maximum venous dilation; RVA, retinal vessel analysis; tMAD, time to maximum arterial dilation; tMAD_30_, time to 30% of maximum arterial dilation; tMVD, time to maximum venous dilation; tMVD_30_, time to 30% of maximum venous dilation.

Suppl. Table 6. Dependence of RVA parameters on treatment with nimodipine at different times after aSAH.

| **Parameter** | **no nimodipine** | |  | **nimodipine** | | **p-value** |
| --- | --- | --- | --- | --- | --- | --- |
|  | median [q1-q3] | n |  | median [q1-q3] | n |  |
|  |  |  |  |  |  |  |
| **CRAE [MU]** |  |  |  |  |  |  |
| Day 0-4 | 163 [135-188] | 14 |  | 147 [134-171] | 23 | 0.2771 |
| Day 5-15 | 159 [145-171] | 8 |  | 163 [137-174] | 26 | 0.9306 |
| Day 16-23 | 174 [132-175] | 8 |  | 175 | 1 | 0.3173 |
| **CRVE [MU]** |  |  |  |  |  |  |
| Day 0-4 | 189 [172-217] | 14 |  | 199 [171-217] | 23 | 0.8822 |
| Day 5-15 | 206 [175-218] | 8 |  | 199 [178-209] | 26 | 0.4594 |
| Day 16-23 | 203 [174-203] | 8 |  | 215 | 1 | 0.1336 |
| **AVR** |  |  |  |  |  |  |
| Day 0-4 | 0.82 [0.78-0.92] | 14 |  | 0.80 [0.72-0.85] | 23 | 0.1326 |
| Day 5-15 | 0.82 [0.76-0.86] | 8 |  | 0.83 [0.75-0.88] | 26 | 0.9826 |
| Day 16-23 | 0.85 [0.83-0.86] | 8 |  | 0.82 | 1 | 0.6171 |
| **MAD [%]** |  |  |  |  |  |  |
| Day 0-4 | 2.5 [1.2-4.0] | 14 |  | 2.8 [1.5-3.6] | 23 | 0.7965 |
| Day 5-15 | 3.3 [1.9-4.4] | 8 |  | 1.9 [1.4-3.7] | 26 | 0.3489 |
| Day 16-23 | 3.8 [0.9-5.0] | 8 |  | 2.1 [1.2-2.9] | 2 | 0.4334 |
| **tMAD [s]** |  |  |  |  |  |  |
| Day 0-4 | 20.0 [17.0-23.5] | 14 |  | 18.0 [15.5-20.0] | 23 | 0.1891 |
| Day 5-15 | 18.5 [16.6-19.5] | 8 |  | 18.0 [15.0-19.0] | 26 | 0.4985 |
| Day 16-23 | 19.5 [16.8-21.0] | 8 |  | 18.0 [13.5-22.5] | 2 | 1.0000 |
| **tMAD_30_ [s]** |  |  |  |  |  |  |
| Day 0-4 | 4.0 [3.5-6.5] | 14 |  | 5.0 [4.5-7.0] | 23 | 0.3149 |
| Day 5-15 | 4.5 [3.9-8.0] | 8 |  | 5.0 [3.0-7.3] | 26 | 1.0000 |
| Day 16-23 | 4.5 [2.0-5.6] | 8 |  | 2.8 [2.4-3.1] | 2 | 0.4263 |
| **AUC_art_ [%*s]** |  |  |  |  |  |  |
| Day 0-4 | 27.8 [9.9-53.3] | 14 |  | 35.8 [15.2-40.7] | 23 | 0.8975 |
| Day 5-15 | 43.9 [12.7-56.5] | 8 |  | 17.7 [7.3-48.9] | 26 | 0.5139 |
| Day 16-23 | 45.1 [11.3-60.2] | 8 |  | 17.2 [9.4-25.0] | 2 | 0.4334 |
| **MVD [%]** |  |  |  |  |  |  |
| Day 0-4 | 4.3 [2.5-4.7] | 14 |  | 3.5 [2.6-4.4] | 23 | 0.5436 |
| Day 5-15 | 3.7 [2.3-5.0] | 8 |  | 4.4 [3.1-5.8] | 26 | 0.4246 |
| Day 16-23 | 3.6 [2.7-4.1] | 8 |  | 1.9 [1.7-2.2] | 2 | 0.1432 |
| **tMVD [s]** |  |  |  |  |  |  |
| Day 0-4 | 21.5 [20.1-22.8] | 14 |  | 21.0 [18.5-22.0] | 23 | 0.3247 |
| Day 5-15 | 20.8 [20.0-26.3] | 8 |  | 21.0 [19.8-22.0] | 26 | 0.6884 |
| Day 16-23 | 20.0 [19.0-21.8] | 8 |  | 21.3 [18.9-23.6] | 2 | 1.000 |
| **tMVD_30_ [s]** |  |  |  |  |  |  |
| Day 0-4 | 7.5 [5.3-10.4] | 14 |  | 7.0 [5.0-8.0] | 23 | 0.3045 |
| Day 5-15 | 7.5 [5.5-12.0] | 8 |  | 7.0 [6.0-8.3] | 26 | 0.9269 |
| Day 16-23 | 7.0 [6.0-7.8] | 8 |  | 7.8 [5.6-9.9] | 2 | 0.7678 |
| **AUC_ven_ [%*s]** |  |  |  |  |  |  |
| Day 0-4 | 36.3 [15.6-56.2] | 14 |  | 42.2 [27.5-47.3] | 23 | 0.9687 |
| Day 5-15 | 30.4 [6.0-44.9] | 8 |  | 47.4 [32.9-61.4] | 26 | 0.1656 |
| Day 16-23 | 42.1 [22.3-52.7] | 8 |  | 13.8 [12.7-14.8] | 2 | 0.1432 |

AUC_art_, arterial area under the curve during flicker stimulation; AUC_ven_, venous area under the curve during flicker stimulation; AVR, retinal arterio-venous-ratio; CRAE, central retinal arteriolar equivalent; CRVE, central retinal venular equivalent; DCI, delayed cerebral ischemia; MAD, maximum arterial dilation; MVD, maximum venous dilation; RVA, retinal vessel analysis; tMAD, time to maximum arterial dilation; tMAD_30_, time to 30% of maximum arterial dilation; tMVD, time to maximum venous dilation; tMVD_30_, time to 30% of maximum venous dilation.
